# DALY-Based Health Risk Assessment of Construction Noise in Beijing, China

**DOI:** 10.3390/ijerph13111045

**Published:** 2016-10-26

**Authors:** Jun Xiao, Xiaodong Li, Zhihui Zhang

**Affiliations:** School of Civil Engineering, Tsinghua University, Beijing 100084, China; xiaoj13@mails.tsinghua.edu.cn (J.X.); zhzhg@tsinghua.edu.cn (Z.Z.)

**Keywords:** construction noise, disability-adjusted life years, social cost, health risk

## Abstract

Noise produced by construction activities has become the second most serious acoustic polluting element in China. To provide industry practitioners with a better understanding of the health risks of construction noise and to aid in creating environmentally friendly construction plans during early construction stages, we developed a quantitative model to assess the health impairment risks (HIA) associated with construction noise for individuals living adjacent to construction sites. This model classifies noise-induced health impairments into four categories: cardiovascular disease, cognitive impairment, sleep disturbance, and annoyance, and uses disability-adjusted life years (DALYs) as an indicator of damage. Furthermore, the value of a statistical life (VSL) is used to transform DALYs into a monetary value based on the affected demographic characteristics, thereby offering policy makers a reliable theoretical foundation for establishing reasonable standards to compensate residents suffering from construction noise. A practical earthwork project in Beijing is used as a case study to demonstrate the applicability of the proposed model. The results indicate that construction noise could bring significant health risks to the neighboring resident community, with an estimated 34.51 DALYs of health damage and 20.47 million yuan in social costs. In particular, people aged 45–54 are most vulnerable to construction noise, with the greatest health risks being caused by sleep disturbance.

## 1. Introduction

Noise pollution is a negative externality of construction activities, especially earthwork and concrete construction. People respond differently to noise pollution; however, when noise levels reach a certain threshold, people tend to be affected negatively [1]. In China, 42.1% of environmental complaints are associated with acoustic pollution, 25.6% of which are attributed to construction noise, and acoustic pollution is becoming a more serious problem as rapid urbanization occurs [2]. Given that the typical projected goals for population urbanization levels increase from 52.6% in 2012 to approximately 60% by 2020, vast construction projects underway to meet the needs of immigrants’ daily lives are creating pervasive environmental pollution and insufferable noise for the neighboring communities. In addition, the impacts of construction noise may be growing more serious because the increasing population density means that more individuals are becoming more exposed to construction noise. Thus, it is critical to take steps to balance the desire for urbanization with sustainable development.

Many measures have been taken to mitigate construction noise pollution. One common method is to reduce noise emissions at the source by using quiet construction technology and equipment and limiting the duration of noisy activities. When source control is unable to reduce noise levels at receptors to below critical levels, the next most common measure is to place obstacles, such as soundproof barriers and enclosures, along the transmission path [3,4]. In addition, some novel techniques derived from industry, such as active noise control techniques [5] and off-site construction [6], can be introduced as supplemental measures to overcome the limitations of the above-mentioned method. However, these above-mentioned measures have high investment costs. For noise control to be successfully implemented in practice, governments must not only implement a strict regulatory system but also develop economic, social and environmental criteria that make such investments worthwhile.

As economic leverage for motivating enterprises to take steps to consciously reduce their pollution, a noise pollution discharge and compensation fee rule has been enforced by the Chinese government in the first national law against noise pollution [7]. In most cities, the discharge fee is intended to fund activities related to noise abatement. A contractor must pay the noise discharge fees to the environmental protection department if the environmental noise boundary of a construction site exceeds the standard limits [8]. The amount of this fee depends on the degree of the violation, ranging from 350 yuan per month for 1 dB above the limit to 11,200 yuan per month for 16 dB or more above the limit [9]. In Beijing, in addition to the discharge fee, a contractor who violates standard limits [8] must compensate the neighboring community by household because of the noise nuisance [10]. According to the rule [10], the actual amount of compensation depends on a negotiation between the contractor and neighbors and is limited to 30–60 yuan per month. These current rules are easy to follow; however, they have three theoretical and operational drawbacks that make it difficult to provide persuasive judgments in their support. First, it is illogical to use the environmental noise level of a construction site boundary as a criterion to determine whether construction noise is a nuisance to neighboring communities, as the noise level at a receptor is not always consistent with that at a site boundary due to propagation attenuation [8,11]. Second, neither rule fully incorporates the health risks caused by noise pollution. Although the noise disturbance compensation rule [10] admits that construction noise can disturb people’s lives, it does not expound upon the exposure–response relationship or even provide a disturbance-grading reference. It is arbitrary to weigh a noise nuisance based on an approximate noise level at a construction site boundary and negotiation. In addition, these rules were enacted many years ago. As environmental awareness and pollution-prevention costs are increasing, the negative externality of construction noise valued by those rules can neither offset people’s losses nor force enterprises to lower noise emissions. Thus, objective pollution assessment methods, as well as convenient estimation methods, are crucial for the economic leverage function of these rules.

Researchers worldwide have used two methods for studies of construction noise emission estimation: the first examines noise data from equipment [3,4,12,13,14,15] and the second from construction sites [16]. Taking advantage of such estimation methods, individual exposure levels can be plausibly measured during the planning phase. However, most studies related to the environmental impacts of construction noise pollution still anchor estimates to noise exposure levels and to the number of communities affected [16,17,18]. A few studies have targeted the negative externality of construction noise. In a study by Gilchrist [19], three sources of social cost (productivity reduction, property damage, and health cost) associated with construction noise are outlined and valued by current bid evaluation methods. Nevertheless, the impacts of construction noise on these three sources were not presented in detail. Moreover, Hong et al. [20] used official standards to calculate the environmental cost of construction noise, but the details of this standard are beyond the scope of this article. Thus, current estimates give no further reference regarding how pollution discharge and compensation fee rules for construction noise are developed and implemented.

Researchers have extended the environmental assessment of transportation noise pollution to social cost valuation using revealed [21,22,23,24,25] and stated preference methods [26,27,28,29,30]. The most common end points for valuation are typically expressed in the form of the Noise Depreciation Index (NDI) or the willingness to pay (WTP), which represent, respectively, the average decrease in house value caused by a 1-decibel increase in the noise level or the amount of money people are willing to pay (WTP) for a 1-decibel decrease in the noise level. These measurements can be directly applied to negotiate transportation-noise charge schemes. However, the models proposed by these studies are not suitable for the assessment of construction noise for two reasons. First, house prices are not vulnerable to construction noise because construction projects usually last 2 years or less. Additionally, the house prices of neighbors will go up if the building being constructed has a public service function. Second, it is questionable that people understand the associated health risks and will make corresponding choices when they value environmental goods [31]. Hence, health risks are apparently more appropriate for deriving the social costs of construction noise.

It has long been recognized that noise has harmful impacts on health [32]. Both experimental and epidemiological studies have been conducted over the years, and the results have revealed an increased disease incidence during periods of noise exposure [33]. Disability-adjusted life years (DALYs) are one of the most widely used metrics for assessing the human burden of disease, and this measure has been adopted as an endpoint indicator for health risk assessment of noise [34,35,36,37,38]. The concept of DALYs was developed in the original 1990 Global Burden of Disease (GBD) study to assess disease burden consistently across different diseases, risk factors and regions [39], and the necessary parameters have been further updated by the World Health Organization (WHO) [40]. A DALY measures the gap between the affected health status and ideal health status and equates it to the sum of time lived with disability and the time lost due to premature death. Social preference, for example, values the life year and can be incorporated to make the results more applicable for environmental decision making [41]. As such, the DALY method is well suited for application in health risk assessment of construction noise.

This paper aims to establish a health risk assessment model for construction noise in Beijing based on the concept of DALYs. The model is intended to serve as a tool to assist the government in improving noise pollution-charge fee rules and to assist contractors in predicting health risks due to noise in order to optimize construction plans during pre-construction and to promote green construction. A commercial building in Beijing is used as a case study to demonstrate how the model works.

## 2. Materials and Methods

### 2.1. Health Risk Analysis

It is necessary to conduct a health risk analysis of construction noise due to the lack of a convenient reference. This section was created to summarize available information on the exposure–response relationships between construction noise and specific health impairments and to describe the background prevalence of disease and the disability weights (DWs) of the outcomes so that DALYs could be applied to assess the health risks of construction noise. Based on the currently available scientific evidence supporting a causal association, the health impairments considered in this study include cardiovascular disease (CVD), cognitive impairment, sleep disturbance and annoyance [33]. Notably, tinnitus was excluded because there are no empirical data to propose that an adverse effect is associated with noise-induced tinnitus [33].

#### 2.1.1. CVD

Noise is considered a general stressor that leads to CVD via arousal of the autonomic nervous system and the endocrine system, which are associated with changes in physiological functions and metabolism, including blood pressure, heart rate, and other functions [32,33]. Recent epidemiological studies have increased understanding of the linkage between community noise and cardiovascular disease (CVD) [42,43], including hypertension [44,45], myocardial infarction [46,47,48] and stroke [49]. However, most CVDs, except myocardial infarction, have only been linked with construction noise by a biological reaction model [50,51,52,53]. An exposure–response relationship with myocardial infarction was demonstrated in a study by Babisch et al. [46]. In that study, the annoyance induced by construction noise had an odds ratio similar to that induced by road traffic noise with respect to myocardial infarction. Hence, our model only considers myocardial infarction when calculating DALYs due to CVD and assumes that construction noise and road traffic noise have the same odds ratio as myocardial infarction at a different sound level. The detailed data shown in Table 1 was originated from local statistics [54] and meta-analysis research on noise exposure–response relationships [47]. Based on six studies and over 17,000 samples, this referenced meta-analysis [47] provided reliable results, and it has also been cited by a 2011 WHO report [33]. Thereafter, Babisch et al. continued their study including more data from five additional studies [48]. However, three of the studies provided information on the overall exposure–response relationship with respect to CVD [55], ischemic heart disease [56] and coronary heart disease [57], which are more common than myocardial infarction. Hence, the present paper relies on the results of previous research [47] to assess myocardial infarction.

The categorical approach of Babisch’s work [47] is noise level-oriented. Relative risks from different studies referring to the same noise category were pooled to derive an exposure–response relationship. The results indicated that exposure to noise increases the incidence of myocardial infarction in people aged 25–74 and has no effects on myocardial infarction incidence in women. To infer the probability risk of myocardial infarction caused by exposure to construction noise, the background prevalence of myocardial infarction in Beijing is also cited. The details are as follows (Table 1):

#### 2.1.2. Cognitive Impairment

The negative effects of noise on learning and memory in children have been studied for many years. Noise can reduce the clarity of a teacher’s voice [59,60], and exposure to noise increases the time required for children to process information [61,62]. The mechanism of this effect is similar to that of CVD. Acute and chronic noise increases the workload of cortical and sub-cortical brain structures, interrupting the original biological response [63]. Children residing in noisier areas of communities suffer psychological distress, as shown by increased systolic blood pressure, greater heart rate reactivity and higher overnight cortisol levels [64,65].

**Figure 1:**
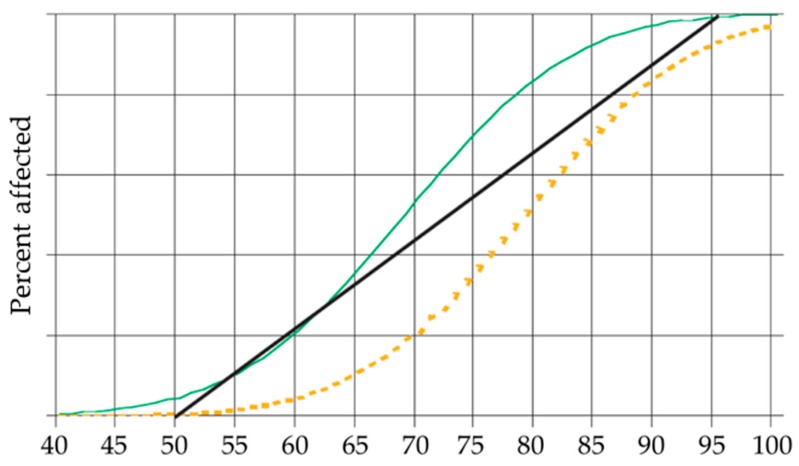
Hypothetical exposure–risk curves and estimated percentages of affected people [33].

Evidence from recent, well-controlled epidemiological studies [66,67,68] with representative samples of children aged 5–19 has made it possible to quantify the magnitude of noise-induced cognitive impairment in children. Based on the results of these studies, the WHO performed a meta-analysis that created an approximate curve (Figure 1) to represent the exposure–response relationship between noise and impairment [33]. This curve assumes that 100% of children exposed to noise at 95 L_dn_, dB(A) suffer from cognitive impairment and that no children are affected at an exposure level of 50 L_dn_, dB(A). The approximate probability of a child developing noise-induced cognitive impairment (NICI) is shown in Table 2.

NICI is not an outcome of a clinical diagnosis [33]. In 2002, WHO have suggested DWs for cognitive impairment and contemporaneous cognitive deficit of different diseases (Japanese encephalitis; Ascariasis; Trichuriasis; Hookworm disease; Iron-deficiency anaemia) in the WPRO B1 (mainly China) region [58]. In 2011, WHO [33] gave a conservative DW of 0.006 in estimates of NICI.

#### 2.1.3. Sleep Disturbance

It is obvious that exposure to noise over a certain threshold during sleeping may cause a person to awaken. Complete sleep cycles are vital to maintaining performance during the day, as well as general good health. Humans perceive, evaluate and react to acoustic information even while asleep. Acute and chronic noise exposure induces the auditory system to continuously engage in these actions and results in sleep structure changes and an increased heart rate. Thus, sleep is disrupted, and its restorative power is decreased.

Sleep disturbance is the most pervasive complaint in China [2], especially in metropolises such as Beijing, Shanghai and Shenzhen, where the traffic conditions make contractors favor arranging material transportation and work at night. Researchers worldwide [69,70,71] have investigated the adverse effects of construction noise exposure on sleep. For example, Sun [69] provided direct evidence in support of health risk assessment for estimating construction noise-induced sleep disturbance in China. In his study [69], 98% of respondents confessed that construction noise reduced their sleep quality, 95% of respondents admitted being awakened by construction noise, and 40% of them reported being awakened frequently. By analyzing the results of noise monitoring and the questionnaire survey, the threshold value of construction noise that leads to sleep disturbance was found to be 52 dB(A) [69]. However, the study did not address the exposure–response relationship between construction noise and sleep disturbance. In contrast, road-traffic noise has been well studied. Miedema [72,73] evaluated 43 data sets obtained from 36 field studies; the data were presented as a synthesis curve that can be seen as the exposure–response relationship between road traffic and sleep disturbance. The responses were gathered from people aged 12–98 years through self-reporting, and the questions addressed problems that included waking up or being disturbed by noise. These two analyses yielded very similar curves and included 95% confidence intervals that considered the variations across individuals and studies. Because construction noise at night in China mainly originates from engines of motor vehicles and heavy equipment, its character could be similar to road traffic noise. Hence, this study assumed that construction noise has the same health risk factor as road traffic noise. The risk factor can be calculated by the following Equation (1) [72]:

(1) $$R=20.8-1.05\times (L_{\mathrm{night}})+0.01486\times {\left(L_{\mathrm{night}}\right)}^{2}$$

where $L_{\mathrm{night}}$ represents the sound level from 11 pm to 7 am in dB(A). The threshold value was found to be 52 dB(A) [69].

Thus far, no DW is available for evaluating noise-induced sleep disturbance in the WPRO B1 (mainly China) region. The only suggested interval that has been published is in the Night Noise Guidelines for Europe [74]. In this report, the median of an interval (0.04, 0.1) was chosen as the DW for noise-induced sleep disturbance. With the assumption that the human response to noise is mainly founded on biological, racial, traditional and other subjective mechanisms, the present study uses a DW of 0.07 to calculate the DALY in Beijing.

#### 2.1.4. Annoyance

Annoyance is widely accepted as a basis for evaluating the impact of noise on an exposed population [33]. People who suffer annoyance may experience multiple negative physical and mental reactions, such as tiredness, stomach discomfort, anger, disappointment and anxiety. Because health is defined as a state of not only physical but also mental and social well-being [32], it is reasonable to consider noise-induced annoyance as a health risk.

Transportation noise is considered the most common source of environmental noise that leads to annoyance. Based on the results of several high-quality papers [75,76,77], the European Commission has created synthesis curves of dose–response relationships for noise annoyance from aircraft, road traffic and railway noise, with their 95% confidence intervals taking into account the variation among individuals and studies [78].

Several studies have shown that construction noise is also a significant noise source that leads to people feeling annoyed [4,69,70,79,80,81]. In a study by Sun [69], field noise levels around a construction plant were measured, and self-reported responses of annoyance were collected. Based on these data, the threshold value of annoyance for construction noise was calculated as 60.3 dB(A); however, no reference to an exposure–response relationship was made. 

Hence, this study assumes that construction noise and road traffic have the same dose–response relationship with annoyance because the annoyance induced by construction noise is primarily caused by inner-combustion engine machines [46,69], which are also the main noise source for road noise. Based on Sun’s study [69] and a report made by the European Commission [78], the function used for risk factor be achieved by Equation (2):

(2) $$R_{\mathrm{daytime}}=9.994\times 10^{-4}{\left(L_{\mathrm{dn}}-60.3\right)}^{3}-1.523\times 10^{-2}{\left(L_{\mathrm{dn}}-60.3\right)}^{2}+0.538\times \left(L_{\mathrm{dn}}-60.3\right)$$

where the unit of $L_{\mathrm{dn}}$ is dB(A).

In the same vein as sleep disturbance, annoyance has no authentic DW. Fortunately, the report by the WHO [33] recommends a conservative DW of 0.02 for estimating DALYs due to annoyance. The age boundary is the same as that for sleep disturbance.

### 2.2. Assessment Model

The health risk of construction noise can be assessed using a model that considers money as a term. First, the DALYs due to construction noise are estimated, using noise level as a dependent variable. Next, the economic value of the DALYs is calculated based on the value of a statistical life (VSL), which is calculated according to WTP.

#### 2.2.1. DALY Estimation Model

In the original method, DALY calculation is performed by statistically analyzing actual data after damage to health has occurred. The following formula is used [33]:

(3) DALY=YLL+YLD

In the Equation (3), YLL represents the number of years of life lost, which is calculated by Equation (4):

(4) $$\mathrm{YLL}=\sum (N_{i}^{m}\times l_{i}^{m}+N_{i}^{f}\times l_{i}^{f})$$

where $N_{i}^{m}\;\mathrm{and}\;N_{i}^{f}$ represent the numbers of deaths of males and females in age group *i* multiplied by the standard life expectancies $l_{i}^{m}$ and $l_{i}^{f}$, of males and females, which is the age at which death occurs. YLD is the number of “years lived with disability”, and is estimated using Equation (5):

(5) YLD=I×DW×D

where I represents the number of incident cases multiplied by a DW and the average duration D of disability in years. DW is associated with each health condition and lies on a scale between 0 (indicating that the health condition is equivalent to full health) and 1 (indicating that the health condition is equivalent to death).

According to this method, noise health risks can be accounted for with $\int_{0}^{\infty }\mathrm{AD}[L,n\left(L\right)]\mathrm{dL}$, where AD(L) represents the DALY loss of each individual exposed to noise level L, and n(L) represents the densities of exposed persons at different noise levels. However, this type of statistical formula cannot satisfy the intention of environmental impact assessment [82]. Hence, in this model, DALYs are calculated using a method modified based on the methodology of the population attributable fraction (PAF), in which the key factor is the relative risk (RR). The expected $N_{i}^{m},\;N_{i}^{f}$ and I can then be derived by n(L)×RR(L, c), which depends not only on the exposed noise level, L, but also on personal characteristics, c, such as age and gender. In addition, data on the distributions of individuals, noise levels and health risks are often available as both discrete and continuous data sets. The formula used for calculating the environmental impact (EI) of noise is expressed as Equation (6):

(6) $$\mathrm{HR}=\underset{L}{\sum }V\left[n\left(L\right)\times \mathrm{RR}\left(L,\;c\right)\times \left(P_{\mathrm{DT}}\times l+\left(1-P_{\mathrm{DT}}\right)\times \mathrm{DW}\times D\right),\;c\right]$$

However, noise around construction sites consists both of background noise and construction noise. Residents could be affected by noise even when there is no construction noise. For example, an environmental noise level above 45 dB(A) could lead to sleep disturbance and annoyance [32], and the standard noise level in most regions of China is above 55 dB(A) [11]. Therefore, to assess construction noise objectively, it is very critical to take the background noise into consideration when calculating DALYs due to construction noise. The formula for this calculation is shown as Equation (7):

(7) $${\mathrm{DALYs}}_{c}{=\mathrm{DALYs}}_{d}-{\mathrm{DALYs}}_{b}=\Delta \;[n\left(L\right)\times R\left(L,\;c\right)\times \left(l+\mathrm{DW}\times D\right)]$$

where ${\mathrm{DALYs}}_{c}$ represents the DALYs caused by construction noise $L_{c}$; ${\mathrm{DALYs}}_{b}$ represents the DALYs caused by the background noise level $L_{b}$; and ${\mathrm{DALYs}}_{d}$ represents the DALYs caused by the environmental noise level $L_{d}$ during construction. Hence, the environmental impact of construction noise, ${\mathrm{EI}}_{c}$, can be expressed as Equation (8):

(8) $${\mathrm{EI}}_{c}\;=\underset{L}{\sum }\Delta V\left[n\left(L\right)\times R\left(L,\;c\right)\times \left(l+\mathrm{DW}\times D\right),c\right]$$

#### 2.2.2. Monetization of Health Risk

In this model, health risks are estimated by DALYs, which consist of both YLL and YLD. YLL influences the duration of life, and YLD influences the quality of life. The key to monetizing the DALY is determining the monetary value of human life. Because life is not a commodity that has a selling price, the economic value of life is not the price of actual life but rather the amount of money people or society are willing to pay for health damage prevention. This amount is the concept of the value of a statistical life (VSL) [83]. VSL is widely used for the valuation of human life in assessments of public safety issues, such as environmental problems and transportation safety [83].

Large quantities of basic data are needed for the estimation of VSL. For research efficiency, the current work calculated the VSL of a citizen in Beijing based on the existing research. Visusi [83] and Aldy [84] summarized numerous methods of estimating VSL and published the VSL for Americans according to the hedonic wage model, which is widely accepted in the field. The VSL is calculated by Equation (9):

(9) VSL=rf×w×t×rv

where rf represents the mortality risk factor (0.00172 according to [85]), w represents the hourly after-tax wage rate, t represents the work hours per year, and rv represents the units of the mortality risk variable (100,000 according to [86,87]).

Because the hourly after-tax wage rate and work hours per year are not provided by state-generated statistics [88], the present study instead uses the per capita disposable income (PCDI) for VSL calculation. After that, the value of a statistical life year (VSLY) for people living in each district of Beijing is calculated based on the VSL and Equation (10), as described below. The results are shown in Table 3.

(10) $$\mathrm{VSLY}=\frac{\mathrm{VSL}\times r}{\left[1-{\left(1+r\right)}^{-n}\right]}$$

where r represents the discount rate (4% according to the European Commission Guideline [89]) and n represents the expected years of remaining life (31.81 according to [84,88]).

Hence, the environmental impact of construction noise, ${\mathrm{EI}}_{c}$, can be expressed as Equation (11):

(11) $${\mathrm{EI}}_{c}={\mathrm{DALYs}}_{c}\times \mathrm{VSLY}$$

In the next section, an example of estimating noise-induced health risks from an earthwork operation is presented to demonstrate the performance of the proposed assessment model. The results are presented for different types of buildings and ages.

## 3. Application to Earthwork

This project is situated in the Da Shilan neighborhood, Xuan Wu District of Beijing, China. This project uses two work platforms, C1 and C2, and the earthwork construction is divided into five parts with a total workload of approximately 400,000 cubic meters. According to the project construction plan, the earthwork construction occurs 24 h a day, is executed by six excavators and eight dump trucks, and lasts 123 days. However, due to changes in site factors and the regulation limits of the Beijing city government, only two excavators are allowed to work, and the earthwork transportation is forbidden from 6:00 am to 11:00 pm, which extends the project schedule to at least 369 days.

Additionally, there are six adjacent buildings, including hotels and residential buildings, within 100 m. According to the “Environmental quality standard for noise” [11], the level of environmental noise in the areas around this project should abide by the limits for the second area, which is 60 dB(A) during the daytime and 50 dB(A) at night.

### 3.1. Data Processing

In this case, the earthwork occurs from 11:00 pm to 6:00 am, and the excavators work all day. Because the environmental impact documents from this project have no relevant data regarding noise, its noise pollution data are collected by field measurement and simulation. The field measurements are performed from 11:00 pm to 6:00 am, and other noise sources that may impact the accuracy of monitoring are virtually nonexistent. The types of data collected include the sound levels of construction noise sources (SL_1_), the local environmental noise levels around the site during construction (SL_2_) and the indoor noise levels in the adjacent buildings (SL_3_). For objectivity and convenience during analysis, data collection is conducted by field monitoring, following the guidelines of the “Emission standard of environmental noise for boundary of construction site” [8], and analysis is performed using the noise-modeling software package SoundPLAN^TM^ Acoustics [90]. Details of the collected data are summarized in Table 4.

**Figure 2:**
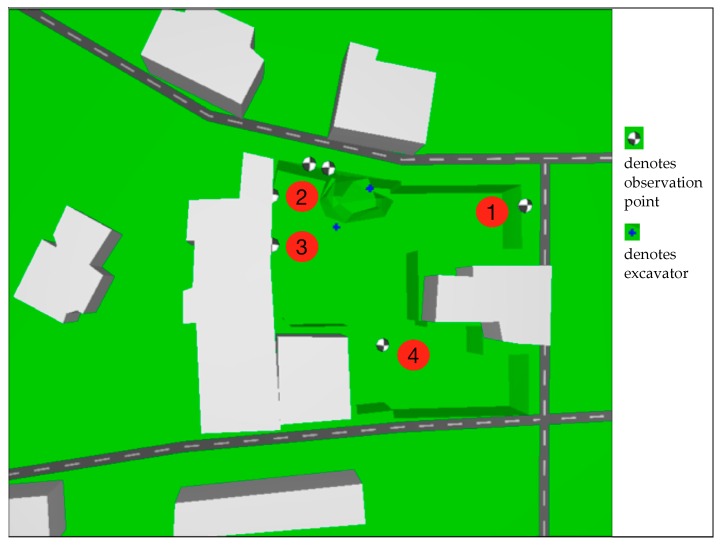
Locations of the observation points and adjacent buildings.

Based on the site conditions and the national standards [8], noise observation points are set to collect noise data. Four of the noise observation points, which are labeled with numbers, are used to measure the noise level of the site, while the other points are set to collect mechanical noise data. The orientation of the observation points and the site terrain are shown in Figure 2.

**Figure 3:**
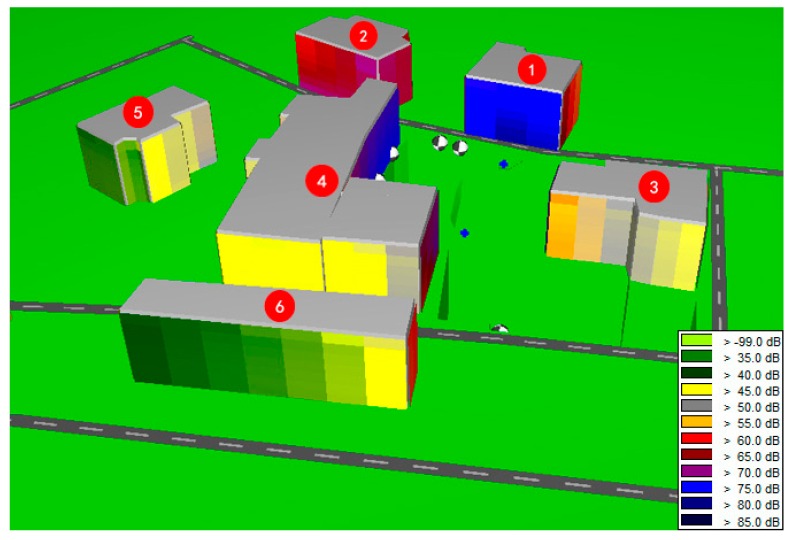
Sound map of the construction site and its vicinity.

The residents living adjacent to the project refused indoor data monitoring; therefore, the environmental noise level before construction was set to 50 dB(A), as authorized by the local government [11]. The environmental noise levels in the adjacent buildings during construction were simulated by SoundPLAN noise-modeling software based on the measured sound power level of the excavator (113 dB) and the environmental noise level before construction. The simulation results are shown in Figure 3 and Table 5. This study first simulates noise levels at the observation points and tests the validity of simulation by comparing the measured data and the simulation data. According to Table 6, the correlation coefficient between the measured data and the simulation data is 1, which indicates a perfect correlation.

**Figure 4:**
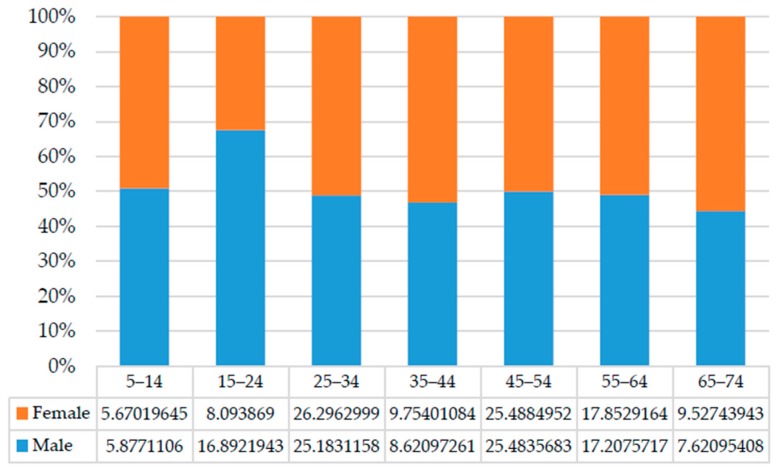
Distribution of age and gender.

After noise data, population information (e.g., size, age and gender) is important for model application. Because the aim of this case study is not to investigate environmental impacts, but rather to present an application of the assessment model, the population-related data used were based on data collected by the local statistical bureau. According to the latest population census statistics of Beijing municipality [91], the per capita floor space for people living in Xuan Wu is 23.54 square meters. Combined with the building area, the population size of each building can therefore be inferred. Then, the expected age and gender distributions for the population in each building can be deduced based on local data and the population size. The Beijing Statistical Association has published census information for the Xi Cheng district that accounts for 10 years and includes the sex ratio [91]. In this case, the distributions of age and gender are assumed to be consistent with that of the Xi Cheng district. For the benefit of data integration, the age interval was set to 10 years, as shown in Figure 4.

### 3.2. Assessment Results

**Figure 5:**
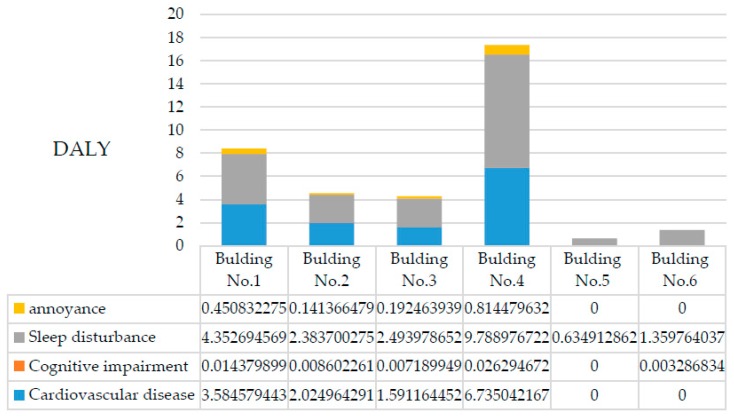
Distribution of environmental impact by building.

**Figure 6:**
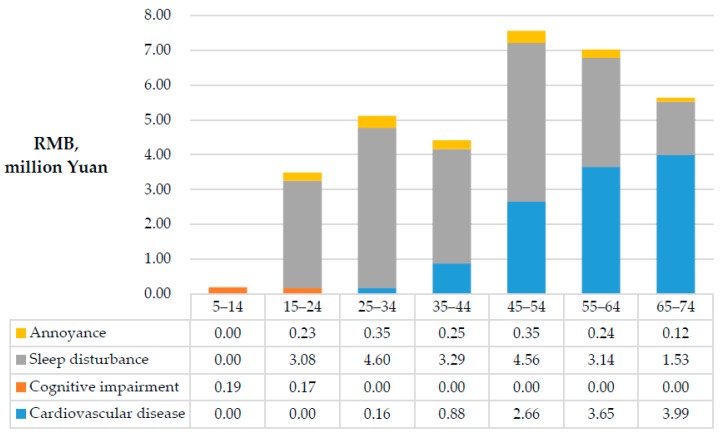
Environmental impacts on age groups.

Based on the above analysis of health risks and VSLY using the presented data on noise levels and population, the DALYs for each building were calculated and are presented in Figure 5 and Figure 6. In the process of DALY calculation, the disability duration of each health impairment, D, is consistent with the duration of the construction period because the evidence showed that these health impairments would disappear once the noise faded away [33].

The total health risk for the neighboring community is 38.0 DALYs, and the corresponding expected social cost of construction noise in this case is almost 19.8 million yuan, of which Building No. 1, Building No. 3 and Building No. 4 are the main contributors. People living in these buildings faced significantly more health risk caused by construction noise for two reasons: (1) they are closer to the noise sources; and (2) there are no barriers to block the noise pathway. People living in Building No. 5 suffered the lowest DALYs because Building No. 4 obstructed the pathway between the noise sources and Building No. 5, where the residents were only slightly influenced by sleep disturbance.

## 4. Discussion

### 4.1. Impact of Noise on Human Health

The present study clearly shows that noise is an important reason for the environmental impact of construction projects, as revealed by the higher health risk faced by the communities neighboring the studied construction site. Among the health risk categories assessed, sleep disturbance formed the largest proportion (more than 55%) and was pervasive in each building. This conclusion is consistent with the fact that most complaints associated with construction noise occurred at night. However, as another widely accepted basis for evaluating the impact of noise, annoyance was not found to have a strong impact, which is likely because its threshold in China is set at 60 dB(A), which is over 10 units higher than that in Europe. Thus, lowering noise emissions at night, developing low-noise equipment and avoiding performing noisy work during nighttime hours represent the most efficient ways to improve a building’s environmental performance, as well as to meet policy goals. The second most widespread health risk in this case was cognitive impairment, which is likely because noise exposure easily challenges a juvenile’s attention. Because only people aged between 5 and 19 years have been shown to be sensitive to NICI, this health risk is only responsible for approximately 0.36 DALYs, which is the smallest calculated health risk. However, cognitive impairment has a negative effect on learning, which is very important for children’s futures. Thus, both the government and contractors should pay special attention to this factor. Compared with the aforementioned risks, CVD only occurred in four buildings but caused approximately 13.93 DALYs, which accounted for 40% of the estimated environmental impact of construction noise. Due to its lethality, which leads to YLL, and the increase in mortality with age, CVD was identified as the most serious health risk for older individuals.

### 4.2. Methodological Limitations

In a study reported by Gilchrist and Allouche [19], property damage was assessed to quantify the social cost of construction noise pollution. As a quiet acoustic climate is critical to recreation, the value that non-residents attach to the amenity may also be relevant, especially in areas that attract large numbers of tourists or where employees working close by go out for lunch [21]. In the present research, Building No. 3, a hotel located on Qianmen Street, a tourist hot spot in Beijing, went from 80% occupancy to 20% occupancy in the second month after the construction began. Due to the absence of actual financial data, the economic loss experienced by this hotel was deduced as 665,118 yuan based on room price (218 RMB per day), number of rooms (15) and duration of construction (at least 369 days). Such costs suffered by businesses cannot be reflected by the model proposed in the present study. Hence, further studies aiming to quantify the social costs of construction noise should integrate property damage and health risk. Another limitation of our study is that the exposure–response factors used were derived from noise with little variation in roughness. The annoyance induced by concrete breaker noise increases as roughness increases [1]. Therefore, some of the risk factors used may have led us to underestimate the severity of the health risk induced by the construction noise produced during the demolition stage. Note, however, that this study focused on applying the DALY concept to quantify the health risks resulting from construction noise. Thus, at best, we can only propose this as a reasonable assessment model. As concern regarding the health impairments caused by construction noise [1,69,70,71] grows, this limitation is expected to be resolved by future studies.

## 5. Conclusions

During urbanization, numerous construction projects occur, which benefit city development and have an environmental impact on the surroundings. As a common source of environmental noise, construction noise not only deteriorates the acoustic climate quality but also threatens resident health, leading to many complaints. To prevent construction noise pollution, some municipal governments, such as that in Beijing, have decided to impose noise discharge fees, which incentivize contractors to reduce emissions. However, these rules are not adequate for policy purposes because thorough consideration of factors that are related to noise level, impact mechanisms and individual characteristics is lacking.

Accordingly, the present study proposes a health risk assessment model for construction noise and establishes an evaluation index for construction projects in Beijing, which has been adopted by the local government to revise noise discharge regulations. Impact assessment is performed using the DALY metric. Four well-accepted health impairments related to noise are thoroughly analyzed. Considering the mechanisms underlying the different health impairments, the related DALYs were assessed by noise level and based on other key factors, such as age, gender and local disease incidence. VSLY was invoked as a monetary index to quantify the social cost of construction noise. Based on the availability of data, the VSLY of individuals living in each district of Beijing are presented, which adds salience to the current noise discharge regulations. In addition, a case study of a construction project in downtown Beijing was used to demonstrate an application of the model.

The case study demonstrated the applicability of the proposed model for assessing the health risk of construction noise. Based on the construction plan, the health risks associated with construction noise from the earthwork case and the corresponding social cost were evaluated using the proposed model. The results of this case study indicate that the health risks caused by construction noise are limited to individuals living in buildings adjacent to construction sites; however, the social cost of the noise is serious. Using this model, administrative departments and contractors can directly predict the environmental impacts of construction noise and take the social cost of construction activity into consideration to implement environmentally friendly construction that helps avoid impairing the health of the people who live and work near a construction site.

However, additional work remains to be conducted. Measurement and assessment efforts based on the methods proposed in the present study should be applied to additional construction sites to gain sufficient empirical data to attain statistical significance. In addition, economic loss should be further investigated to objectively assess the social cost of construction noise. Note that, in principle, property damages evaluated by rental loss, occupancy loss and other kinds of income loss are determined by people’s perceptions of noise nuisance. Hence, simply combing the value of health risks and economic loss would lead to overestimation. Therefore, future improvements should also emphasize strategies and approaches using integrated methodologies.

## Figures and Tables

**Table 1:** Probability risk ^a^ of myocardial infarction caused by construction noise, in percent.

	Sound Level, L_dn_, (dB(A))	<60	60–64	65–69	70–74	75	Disability Weight ^b^, DW
Age							
25–34		0.0105	0.0106	0.0112	0.0121	0.013	0.443
35–44		0.0796	0.0808	0.0850	0.0924	0.1036	
45–54		0.2059	0.2090	0.2197	0.2391	0.2681	
55–64		0.3936	0.3995	0.4200	0.4570	0.5125	
65–74		1.3436	1.3637	1.4336	1.5599	1.7493	

^a^ $R=\mathrm{odds}\;\mathrm{ratio}\times P_{\mathrm{mt}}$, where odds ratio is derived from [47], and $P_{\mathrm{mt}}$ represents the local average mortality level of myocardial infarction [54]; ^b^ The DW for myocardial infarction in the WHO WPRO B1 (mainly China) region is 0.433 [58].

**Table 2:** Health risk of noise-induced cognitive impairment (NICI).

Noise Level L_dn_, (dB(A))	Percentage of Children Who Will Suffer from NICI	Disability Weight, DW	Boundary Condition
<55	0%	0.006	Age: 7–19 years old
55–64	20%		
64–75	50%		
>75	75%		

**Table 3:** VSLY for Beijing citizens (in RMB).

Location	PCDI	VSLY
Dong Cheng	45,052	434,770.61
Xi Cheng	47,392	592,962.95
Chao Yang	44,646	558,605.34
Feng Tai	41,334	517,165.99
Shi Jingshan	41,943	524,785.73
Hai Dian	50,088	626,694.98
Men Tougou	38,023	475,739.16
Fang Shan	35,912	449,326.59
Tong Zhou	37,095	464,128.14
Shun Yi	36,428	455,782.72
Chang Ping	35,517	444,384.39
Da Xing	37,131	464,578.57
Huai Rou	35,771	447,562.41
Ping Gu	36,226	453,255.32
Mi Yun	35,499	444,159.18
Yan Qing	33,778	422,626.24

**Table 4:** Description of the data.

Data Type	Collection Method	Utility
SL_1_	Field monitoring	Forecast the acoustic environment of the adjacent buildings during construction
SL_2_	Field monitoring and acoustics simulation software	Test the validity of simulation
SL_3_	Acoustics simulation software	Represent the acoustic environment in the adjacent buildings during construction

**Table 5:** Simulated noise levels in the surrounding buildings (unit: dB(A)).

Building No.	Noise Level L_Aeq_					
	1st Floor	2nd Floor	3rd Floor	4th Floor	5th Floor	6th Floor
1	80.6	80.0	79.2	78.2	77.2	76.2
2	69.5	68.0	67.4	67.6	67.7	69.2
3	73.0	73.2	72.9	72.6	72.3	72.0
4	75.0	74.8	74.4	74.0	73.6	73.1
5	52.6	52.9	53.0	53.0	52.9	52.8
6	59.3	59.1	59.5	59.6	59.8	59.9

**Table 6:** Description of the noise data (unit: dB(A))

Point No.	Measured Value (L_Aeq_)	Simulation Value (L_Aeq_)	Errors ^a^
1	67.8	66.4	1.4
2	73.0	73.2	−0.2
3	73.9	73.5	0.4
4	68.5	67.0	1.5
Correlation coefficient	1 ^b^		

^a^ Errors equals the measured value minus the simulation value; ^b^ Correlation is significant at the 0.01 level (2-tailed).
